# Breast cancer treatment: When less is more

**DOI:** 10.1371/journal.pmed.1005284

**Published:** 2026-10-26

**Authors:** Katarzyna Starowicz, on behalf of the PLOS Medicine Staff Editors

**Affiliations:** Public Library of Science, Cambridge, United Kingdom

## Abstract

Breast cancer treatment using systemic therapies can cause severe side effects that negatively impact patient quality of life and function, sometimes with limited clinical benefit. In this Editorial, Katarzyna Starowicz discusses how precision oncology tools may help us identify those who can safely reduce therapy to avoid overtreatment.

## Teaser

Breast cancer treatment using systemic therapies has often focussed on remission and survival, but can cause severe side effects that negatively impact patient quality of life and function, sometimes with limited clinical benefit. In the era of precision oncology, one of the greatest opportunities may not be in identifying more patients for treatment, but rather in identifying those who can safely receive less to avoid overtreatment.

Advances in breast cancer treatment offer one of the best examples illustrating the success of modern oncology. Over the past decade, early diagnosis and effective therapies have led to ~2% to 2.5% annual breast cancer mortality reductions in several high-income countries, albeit not worldwide [1]. Improvements in radiotherapy, chemotherapy, HER2-targeting agents, endocrine therapy, immunotherapy and antibody-drug conjugates have transformed outcomes for many patients. However, these remarkable advances have also created a paradox: while many patients benefit from systemic treatments, acute and long-term toxic effects are common and may negatively impact the quality of life of patients [2]. In general, approaches have focussed on minimising possible recurrence, justified by the absence of reliable biomarkers to tailor treatment strategies [3]. Nowadays, a personalised approach to cancer care—precision oncology—has significantly improved our ability to characterise a tumour, predict responses to treatment, and estimate possible risks. As a result, the power in precision medicine for breast cancer might not lie in escalating treatment but in withholding it in cases where treatment would have small benefit-to-harm ratios, e.g., due to limited efficacy and harmful side effects. Notably, the limited treatment strategy called watchful waiting, which prioritises managing symptoms and palliative care, and active surveillance, which involves structured monitoring to detect disease progression early and enable timely treatment where appropriate, are increasingly implemented for prostate cancer management to avoid overtreatment [4].

Overtreatment in breast cancer is an unintended consequence of medical progress as more therapies become available. The repercussions for patients often extend well beyond transient adverse events. Surgery may cause chronic pain, restricted shoulder movement, change physical appearance and alter body image. Radiation is painful and can lead to skin damage and tissue fibrosis. Chemotherapies and some biologics are associated with infertility, fatigue, cardiotoxicity (as seen in HER2-targeted treatments or anthracyclines), cognitive impairment, and secondary malignancies; some of these effects may persist long after treatments end. Endocrine therapy such as tamoxifen, usually prescribed long-term (5+ years), can lead to musculoskeletal symptoms, depression, sexual dysfunction, osteoporosis, vasomotor symptoms, and increased risk of endometrial cancer and blood clots [2,5,6]. Thus, to avoid overtreatment, the concept of “de-escalation” (also known as “de-intensification”) is being increasingly implemented, whereby treatment duration and intensity is reduced as much as possible to minimise adverse effects without compromising outcomes.

The clearest evidence for de-escalating treatment in breast cancer comes from genomic risk stratification. Trials, such as TAILORx, RxPONDER, MINDACT, and more recently OPTIMA, are changing the management of therapies for different types of breast cancer, showing that patients with intermediate or low genomic risk (but high clinical risk of recurrence) derive little or no benefit from chemotherapy and can safely avoid it while maintaining good outcomes [7]. These trials illustrate that stratifying patients by genomic risk can reduce rather than increase intervention, a fundamental shift from the concept that molecular diagnostic tools serve only for the identification of potential candidates for novel targeted therapies. First-generation genomic assays, including 21-gene Oncotype DX recurrence score and the 70-gene MammaPrint signature, are among available tools used for treatment de-escalation [8]. Other emerging technologies include the use of circulating tumour DNA (ctDNA) as a biomarker, which can be used to identify “molecular relapse” months before clinical recurrence will be detected by imaging and biopsy, although there is not yet enough evidence to show that earlier detection of relapse can improve patient outcomes or reduce treatment intensity [9].

The use of artificial intelligence (AI) in breast cancer screening, diagnosis and risk prediction is also rapidly expanding. For example, AI-based approaches have been utilised for analysing mammograms in breast cancer screening, such as the Transpara system used in the MASAI trial. Here, AI-supported screening was compared with standard screening using double reading by radiologists; AI-supported screening resulted in similar cancer detection rates, while workload was reduced by 44.3% [10]. Elsewhere, AI tools have been implemented in genomic-based recurrence and treatment benefit prediction (e.g., Oncotype DX), recurrence risk prediction (e.g., PreciseBreast), and digital pathology analysis and treatment response (e.g., ENLIGHT–DeepPT) [8]. While these AI tools have shown impressive performance, most published studies evaluate diagnostic accuracy and not patient outcomes or benefits.

Identifying candidates for treatment de-escalation for breast cancer has important global implications. In many low- and middle- income countries (LMICs), in which breast cancer is the leading cause of death due to cancer among women [11], oncology services, diagnostic infrastructure, qualified personnel availability, and access to specialised (and often expensive) medicines can be limited. Avoiding unnecessary treatment that provides little benefit, by matching patients to appropriate personalised therapeutic regimens, would have clinical and economic value, and could facilitate redirection of resources towards more urgent interventions or other aspects of cancer care. In many LMICs, however, limited access to screening and treatment and delays in diagnosis remain major barriers [12]. Where mammography screening is not feasible, improving symptom awareness, reducing barriers to presentation, and establishing rapid diagnostic pathways may have greater impact on survival. AI-assisted tools may help address some of the infrastructure and workforce constraints, but require local validation and should complement, not divert, resources from essential services. The *Lancet Breast Cancer Commission* [13] and WHO Global Breast Cancer Initiative [14] have advocated for context-specific phased approaches centred on early detection, timely diagnosis and comprehensive treatments that are adapted to local resources and measure inequities and outcomes.

Reducing global mortality of breast cancer and patient burden requires simultaneous progress in early detection, timely diagnosis, and identification of the most suitable treatment regimens using modern tools, while avoiding overtreatment. Small gains in recurrence reduction must be balanced against long-term toxicity, survivorship, financial burden, resource restraints and patient preferences. Doctors and healthcare workers should communicate not only the benefits of treatment but also the possible adverse (including long-term) effects. Patients should be educated that avoiding unnecessary treatment doesn’t mean withholding therapy but rather reflects a commitment to providing the best possible care.

Future research priorities for breast cancer should focus on providing evidence from randomised non-inferiority trials with long term follow-up for evaluation of treatment reduction or omission. Biomarker stratified trials are also needed to establish whether genomic assays (e.g. ctDNA tests) improve clinical benefit rather than only predicting risk. At *PLOS Medicine*, we welcome studies evaluating treatment strategies, including de-escalation and optimisation, as well as precision medicine approaches such as genomic risk stratification tools, particularly where they provide robust evidence on patient-centred outcomes, treatment burden, and quality of life.

Breast cancer is not only life-changing due to diagnosis, but also the impact(s) of treatment. Advances in precision oncology should allow patients to make more informed and educated decisions about their treatment, balancing potential benefits and harms of undergoing therapy, de-escalation, or active surveillance to maximise their chances of remission and survival while preserving or improving their quality of life.

## Abbreviations

AI: artificial intelligence
LMICs: low- and middle- income countries

## References

[1] Kim J, Harper A, McCormack V, Sung H, Houssami N, Morgan E, et al. Global patterns and trends in breast cancer incidence and mortality across 185 countries. Nat Med. 2025;31(4):1154–62. doi: 10.1038/s41591-025-03502-3 39994475

[2] Lustberg MB, Kuderer NM, Desai A, Bergerot C, Lyman GH. Mitigating long-term and delayed adverse events associated with cancer treatment: Implications for survivorship. Nat Rev Clin Oncol. 2023;20(8):527–42. doi: 10.1038/s41571-023-00776-9 37231127 PMC10211308

[3] Harbeck N, Wuerstlein R. Truly personalized therapy: An end to the era of one size fits all. Nat Rev Clin Oncol. 2019;16(2):77–8. doi: 10.1038/s41571-018-0165-1 30644455

[4] Homewood D, Lucas H, Kennedy C, Majer J, Sathianathen N, Corcoran NM. Focus | Clinical. vol. 53. 2024. 10.31128/AJGP-06-23-6866 38697055

[5] Bodelon C, Masters M, Bloodworth DE, Briggs PJ, Rees-Punia E, McCullough LE, et al. Physical health decline after chemotherapy or endocrine therapy in breast cancer survivors. JAMA Netw Open. 2025;8(2):e2462365. doi: 10.1001/jamanetworkopen.2024.62365 40019757 PMC11871543

[6] Davies C, Pan H, Godwin J, Gray R, Arriagada R, Raina V, et al. Long-term effects of continuing adjuvant tamoxifen to 10 years versus stopping at 5 years after diagnosis of oestrogen receptor-positive breast cancer: ATLAS, a randomised trial. Lancet. 2013;381(9869):805–16. doi: 10.1016/S0140-6736(12)61963-1 23219286 PMC3596060

[7] Lobo-Martins S, Luen SJ, Piccart M, Loi S. Tailoring targeted therapies for younger women with ER-positive early-stage breast cancer. Nat Rev Clin Oncol. 2026;23(4):297–315. doi: 10.1038/s41571-026-01120-7 41634433

[8] Polit F, Bahmad HF, Kassab MB, Elajami MK, Recine M, Alghamdi S, et al. Digital pathology and artificial intelligence in breast and gynecologic oncology: From molecular prediction to multimodal integration. Front Oncol. 2026;16:1833926. doi: 10.3389/fonc.2026.1833926 42109656 PMC13152864

[9] Panet F, Papakonstantinou A, Borrell M, Vivancos J, Vivancos A, Oliveira M. Use of ctDNA in early breast cancer: Analytical validity and clinical potential. NPJ Breast Cancer. 2024;10(1):50. doi: 10.1038/s41523-024-00653-3 38898045 PMC11187121

[10] Hernström V, Josefsson V, Sartor H, Schmidt D, Larsson A-M, Hofvind S, et al. Screening performance and characteristics of breast cancer detected in the Mammography Screening with Artificial Intelligence trial (MASAI): A randomised, controlled, parallel-group, non-inferiority, single-blinded, screening accuracy study. Lancet Digit Health. 2025;7(3):e175–83. doi: 10.1016/S2589-7500(24)00267-X 39904652

[11] Sung H, Filho AM, Laversanne M, Ferlay J, Siegel RL, Soerjomataram I. Global cancer statistics 2024: GLOBOCAN estimates of incidence and mortality worldwide for 34 cancers in 186 countries. CA Cancer J Clin. 2026;76. doi: 10.3322/caac.70090 PMC13343830 42417444

[12] Ebrahimoghli R, Aghaei MH, Azami-Aghdash S, Houssami N. Uptake of breast cancer screening practices in low- and middle-income countries: A systematic review and meta-analysis. J Natl Cancer Inst. 2025;117(1):29–39. doi: 10.1093/jnci/djae187 39133184

[13] Coles CE, Earl H, Anderson BO, Barrios CH, Bienz M, Bliss JM. The Lancet Breast Cancer Commission. The Lancet. 2024;403. doi: 10.1016/S0140-6736(24)00747-5 38636533

[14] World Health Organization. Global breast cancer initiative implementation framework: Assessing, strengthening and scaling-up of services for the early detection and management of breast cancer. Executive Summary. 2023. Availablefrom: https://www.who.int/publications/i/item/9789240067134

